# Process Evaluation of a Blended Web-Based Intervention on Return to Work for Sick-Listed Employees with Common Mental Health Problems in the Occupational Health Setting

**DOI:** 10.1007/s10926-016-9643-4

**Published:** 2016-05-05

**Authors:** D. Volker, M. C. Zijlstra-Vlasveld, E. P. M. Brouwers, C. M. van der Feltz-Cornelis

**Affiliations:** 1 Trimbos-institute, Netherlands Institute of Mental Health and Addiction, P.O. Box 725, 3500 AS Utrecht, The Netherlands; 2 Tranzo Department, Tilburg University, Tilburg, The Netherlands; 3 TopClinical Centre for Body, Mind and Health, GGZ Breburg, Tilburg, The Netherlands

**Keywords:** Process evaluation, Feasibility, Occupational health, Common mental disorders, Return to work, eHealth

## Abstract

*Purpose* A blended web-based intervention, “eHealth module embedded in collaborative occupational health care” (ECO), aimed at return to work, was developed and found effective in sick-listed employees with common mental disorders. In order to establish the feasibility of ECO, a process evaluation was conducted. *Methods* Seven process components were investigated: recruitment, reach, dose delivered, dose received, fidelity, satisfaction and context. Quantitative and qualitative methods were used to collect data: an online questionnaire for the employees, website data, telephonic interviews with occupational physicians (OPs) and observations of the researchers. *Results* Recruitment was uncomplicated for the employees, but required several steps for the OPs. Reach was 100 % at the OP level and 76.3 % at the employee level. Dose delivered and received for OPs: 91.6 % received minimally one email message. Dose delivered and received for the employees: finishing of the different modules of ECO varied between 13 and 90 %. Fidelity: the support of the OP to the employee in ECO was lower than anticipated. Satisfaction: both employees and OPs were satisfied with the intervention. However, employees reported a need for more support in ECO. The context showed that OPs had limited time to support the employees and it was impossible for the employee to contact the OP outside their regular contacts. *Conclusion* Feasibility of ECO and satisfaction of employees and OPs with ECO were good. Fidelity of OPs was limited. For further implementation in the occupational health setting, especially contextual barriers regarding time limitation and accessibility of OPs for employees should be addressed.

## Introduction

### Background

Long-term sickness absence is a major public health problem in the Western world and leads to enormous cost [1]. A large part of the costs of sickness absence is caused by common mental disorders [1, 2]. In the treatment of sick-listed employees the focus is mostly on symptom recovery and return to work is often not addressed [3, 4]. However, several studies have shown that focusing on symptoms alone is not enough to achieve return to work (RTW) [5, 6].

Recently, a blended web-based intervention focusing on return to work for sick-listed employees with common mental disorders was developed and found to be effective, both in terms of return to work and in terms of symptom relief, in a randomized controlled trial (RCT) in the occupational health setting [7, 8]. The intervention “eHealth module embedded in collaborative occupational health care” (ECO) combines an eHealth intervention for the sick-listed employees with monitoring the employees’ progress in mental health and a decision aid for the occupational physician (OP) [7, 8].

### Rationale

Implementation of ECO in the occupational health setting seems warranted based on the findings in the RCT; however, implementation of eHealth interventions in routine practice is challenging [9, 10] and a blended eHealth intervention guided by OPs is new in the occupational health setting. It has been argued that a process evaluation may facilitate the interpretation of the results of the RCT and the implementation of the intervention in the future [11–13]. A frequently applied framework for process evaluation is developed by Steckler and Linnan [11], containing the following components: recruitment (procedures used to approach participants), reach (participation rate), dose delivered (completeness), dose received (exposure), fidelity (the extent to which the intervention was delivered as planned) and context. Saunders et al. describe how to develop a process-evaluation plan and extend the framework of Steckler and Linnan with the component satisfaction [12].

### Objective

The aim of this study is to perform a process evaluation following the evaluation plan of Saunders et al., and to investigate the feasibility of the ECO-intervention in the occupational health setting; to report the experiences of the employees and the OPs with the ECO-intervention and to give recommendations for further implementation of the ECO-intervention.

## Methods

### Design

In this mixed method study, data for this process evaluation were gathered alongside a RCT [7, 8]. This study was conducted between 2011 and 2013. In this process evaluation seven process components were defined: recruitment, reach, dose delivered, dose received, satisfaction, fidelity and context [11–15]. Quantitative and qualitative methods were used to collect data on the process components: an online questionnaire for the employees at 3 months, user statistics, telephone interviews with OPs, and observations of the researchers.

### Study Population

#### Occupational Physicians

In the Netherlands all sick-listed employees have to visit an OP within the first 6 weeks of sickness absence. The participating OPs in the current study were employed by Arbo Vitale (a large occupational health service) and GGz Breburg (a large mental health service). In total, 32 OPs were randomized in the intervention condition of the RCT and received training in the ECO-intervention. For this process evaluation a selection of OPs was interviewed by telephone in 2012, halfway through the inclusion period of the RCT. At that moment 12 OPs had guided or were guiding one or more employees in the ECO-intervention. These OPs were asked if they wanted to participate in the interview and 11 OPs responded positively.

#### Sick-Listed Employees

The participating employees were sick-listed employees visiting their OP at Arbo Vitale and sick-listed employees of GGz Breburg visiting their OP. Inclusion criteria and recruitment procedure are extensively described elsewhere [7, 8]. In total, 220 employees participated in the RCT, of which 131 were randomized in the intervention group and 89 in the control group [8]. The 131 employees in the intervention group were approached for the process evaluation. The 89 employees in the control group were approached only with respect to their satisfaction with the occupational health service and OP in general.

### Intervention Protocol

The ECO-intervention is also extensively described in Volker et al. [8]. For the purpose of this process evaluation, the intervention is summarized here.

**Fig. 1:**
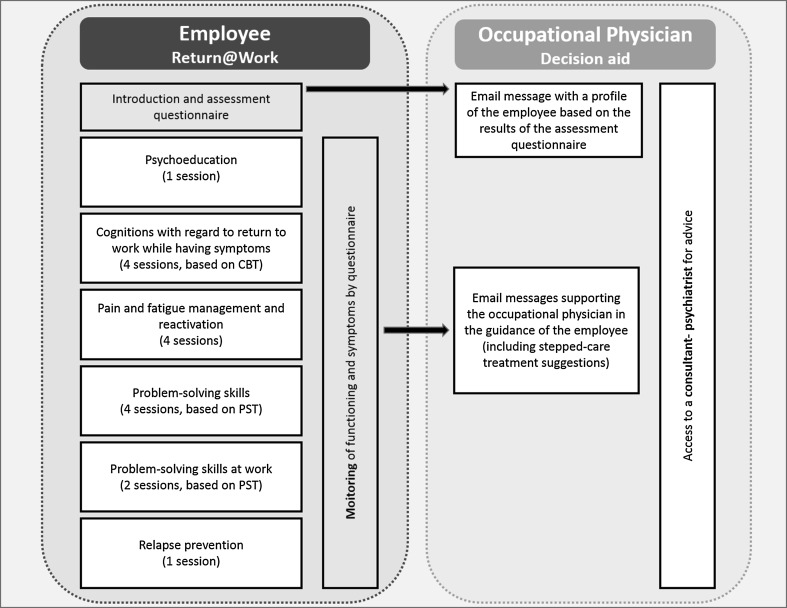
Overview of the ECO intervention

Figure 1 shows a schematic overview of the intervention. The ECO-intervention included two elements: an eHealth module for the employees and a decision aid for the OPs. The first element, the eHealth module “Return@Work,” included the following five modules (see Fig. 1). The content of Return@Work was tailor made to the individual employee, depending on the symptoms and cognitions about RTW reported by the employee at the assessment questionnaire, therefore the duration of Return@Work varied between six and sixteen sessions. Employees were advised to do at least one session per week. The employees worked through Return@Work individually, however the OPs were instructed to inquire about the employee’s progress in Return@Work in their regular contact with the employees and to support the employee if necessary.

The second element of the ECO-intervention is the decision aid by email for the OP. The OPs received automated emails that were based on a decision aid with principles of stepped, collaborative care. The decision aid supported the OPs in the sickness guidance of the employees in the monitoring of symptoms, functioning and RTW. Furthermore, the decision aid gave the OP access to a consultant psychiatrist who, if needed, gave consultation advice in case of stagnation or problems regarding carrying out the suggestions in the decision aid [8]. The OPs received a half day training in the ECO-intervention. In the training, OPs were taught the background and content of Return@Work, and were instructed how to guide employees through Return@Work and how to work with the decision aid. They were taught the basic principles of problem solving treatment and cognitive behavioral therapy and how to apply these principles to guide the employee.

If employees agreed to participate in the RCT and were randomized to the intervention group, the following procedure was applied. First, the researchers created an account for the employee for the eHealth module, Return@Work. Next, the employee received an automatic email containing a link to the Return@Work website, a username and a password. When the employee logged into Return@Work he started with an assessment questionnaire, which included questions about symptoms, functioning and cognitions about RTW. The OP of the participating employee received an automated email after the participant ended the assessment questionnaire. The employee was asked for permission to send monitor results from Return@Work to the OP. After every six sessions in Return@Work the employee had to fill out a monitor questionnaire and the OP received an email based upon this questionnaire containing information about the employee’s progress. All employees signed informed consent prior to the start of the intervention. The study protocol was approved by the Medical Ethics Committee of the University Medical Center Utrecht in the Netherlands in 2011.

### Data Collection of the Process Elements

The intervention evaluated in this process evaluation was implemented in the context of a RCT [7, 8]. This process evaluation focusses on the ECO-intervention and not on the RCT. When possible the process elements will be described at the level of the OP and the employee.

#### Recruitment

Recruitment refers to the procedures used to approach participants for the intervention. The recruitment of the OPs and employees will be described.

#### Reach

Reach is the proportion of the intended audience that participated in the intervention. In the present study we defined reach for the OPs as the proportion of OPs that participated in the training of the ECO-intervention.

We defined reach for the employees as the proportion of employees that started Return@Work divided by the number of employees that received an account for Return@Work.

#### Dose Delivered and Received

Dose delivered is defined as the amount of intended interventions that is actually delivered to the participants. Dose received is defined as the extent to which participants actively engaged with the intervention. In the current process evaluation we combined dose delivered and received and defined this at the OP level as the number of OPs that received email messages from the decision aid of the ECO intervention.

Dose delivered and received for the employees was evaluated by reporting the number of employees that have started and finished the different modules of Return@Work. Furthermore, the percentage of employees who discussed Return@Work with their OP will also be reported. Part of this information was also reported in the article about the effectiveness of the ECO-intervention [8].

#### Fidelity

The process component fidelity refers to the extent to which the intervention was implemented and delivered as planned. The fidelity in this study was evaluated by conducting telephone interviews with 11 OPs. Prior to the interviews, a topic list was developed that consisted of questions related to practical issues, content of email messages, the guidance of the employee through the ECO-intervention, adherence of the employees, results of the ECO-intervention and eHealth in general. The interviews lasted for about 15–30 min, were digitally recorded and transcribed verbatim. Furthermore the number of OPs that have contacted the consultant psychiatrist was reported.

#### Satisfaction

At the level of the OPs during the telephone interviews held with OPs, their satisfaction with the ECO-intervention in general, the email messages from the decision aid and the consultant-psychiatrist were evaluated.

The satisfaction of the employees was measured in two ways. First, to assess the opinion of the employees about the ECO-intervention, the employees were asked to give comments about Return@Work in an online questionnaire 3 months after baseline. In these open-ended questions the employees were asked for positive and negative feedback and suggestions for improvement. The answers of the employees were clustered together by theme by the researchers. Secondarily, the satisfaction of the participating employees with the occupational health service and OP in general was measured 3 months after baseline with the Patient Satisfaction with Occupational Health Questionnaire (PSOHQ) in the ECO-group and control group [16]. The questionnaire contains five subscales, namely: being taken seriously as a patient, attitude towards occupational health services (OHS), trust and confidentiality, expectations and comfort and access.

#### Context

Context refers to aspects of the environment that may have influenced the implementation of the intervention. The process component context was assessed by the telephone interviews with the OPs (see fidelity) and by field notes of the researchers during the study, as observations about how the context could have influenced the intervention.

### Data Analysis

Data from the telephone interviews with the OPs and the answers of the employees to the open-ended questions were categorized and coded through thematic coding, using the qualitative data analysis software program MaxQDA [17]. The data of the PSOHQ was compared between the care as usual (CAU) and ECO group using independent *t* tests. These analyses were performed in SPSS [18].

## Results

### Recruitment

*OP Level* The OPs randomized in the intervention condition were expected to follow a half day training the ECO-intervention. All OPs received access to a joint mailbox where the email messages from the decision-aid were sent. When an email was sent to the joint mailbox of the OPs, the administrative assistant received an email message with the assignment to alert the OP at the email message in the joint mailbox.

*Employee Level* The employees participating in the RCT and randomized to the intervention group received an automated email with a login account for Return@Work.

### Reach

*OP Level* All OPs who were randomized in the intervention group received training in the ECO-intervention; this resulted in a dose delivered of 100 % for the OPs (32/32).

*Employee Level* All 131 employees randomized in the ECO group received an account which provided them access to Return@Work, and 100 employees actually started with Return@Work. This resulted in a reach of 76.3 % (100/131) for the employees.

### Dose Delivered and Received

*OP Level* The OPs received an automated email message from the decision aid when the participating employee ended the assessment questionnaire in Return@Work, when the employee filled out the monitor questionnaire in Return@Work (after six and twelve sessions) and when the employee filled out the end questionnaire in Return@Work. In total, four OPs received all four email messages (12.5 %), four OPs received three email messages (12.5 %), 10 OPs received two email messages (31.3 %), 11 OPs received one email message (34.4 %) and three OPs (9.4 %) did not receive email messages.

*Employee Level* Table 1 presents the dose received and the number of employees that started and finished the different modules of Return@Work. The module “relapse prevention” was offered to the employees only if they reported that they had (at least partly) returned to their work in the meantime. Furthermore, 29 % (20/69) of the employees reported at the 3-month questionnaire that they discussed Return@Work at least once with their OP.

**Table 1:** Number of employees that started and finished the modules of Return@Work

Modules	Number of employees started	Number of employees finished
Introduction and assessment questionnaire	100	90 (90 %)
Psychoeducation	69	65 (94 %)
Cognitions with regard to RTW while having symptoms^a^	59	33 (56 %)
Pain and fatigue management^a^	31	28 (90 %)
Problem-solving skills	30	4 (13 %)
Relapse prevention	0	0

^a^These modules were not offered to all employees, depending on their scores at the assessment questionnaire

### Fidelity

In the training that the OPs received, they were instructed to inquire about the employee’s progress in Return@Work at the regular consultations. In the interviews the OPs reported that, due to their unawareness of the participation of the employees in Return@Work, they did not do this consistently. Several OPs reported that they sometimes did not notice the emails from the decision aid in the joint mailbox or that at the time the employee was having the next consultation the OP had forgotten that he had received an email about the employee some time ago. As described by “recruitment”, the administrative assistants were ordered to put a remark in the record of the employee to alert the OP to an email message from the decision aid in the joint mailbox. The OPs reported that the administrative assistants did not do this. In consequence of the remarks of the OPs, the researchers adjusted the infrastructure of the ECO-intervention by letting the emails from the decision aid also be sent to the direct mailbox of the OPs as well as the joint mailbox.

Furthermore, the OPs reported that the employees rarely asked questions about Return@Work to their OP. During this study the psychiatrist was consulted by only one OP. This was lower than expected. In the training of the ECO-intervention the OPs were told that they could consult a psychiatrist in case of stagnation. Unfortunately, we do not have information about how many employees experienced stagnation.

### Satisfaction

*OP Level* In the interviews with the OPs, they reported that, in general, they were satisfied with the ECO-intervention. The OPs stated that the email messages from the decision aid supported them in the guidance of the employees. They reported that the email messages gave them sufficient information and the lay-out was visually attractive. Furthermore, they experienced having the opportunity to contact a psychiatrist when necessary as comforting.

*Employee Level* The open-ended questions to assess the opinion of the employees about Return@Work at the 3-month questionnaire were completed by 61 employees. The most frequently reported positive aspect of R@W, as reported by the employees, was that it gave them more insight and understanding of their problems and symptoms. The information about negative and positive thoughts/cognitions of the module “cognitions with regard to RTW while having symptoms” was most frequently mentioned as a positive point of Return@Work. Other positive aspects mentioned by employees were that they recognize their problems in the texts, the focus on return to work, learning problem-solving skills, receiving advice about physical complaints and that Return@Work gave food for thought. The most frequently reported negative aspect of Return@Work was that there was too little guidance/contact, feedback and personal attention. Another frequently mentioned negative aspect was that Return@Work was too general or not applicable to their situation or disease/symptoms. Suggestions for improvement reported by the employees were more contact (with OP or the researchers), sending reminders to continue Return@Work and not repeatedly asking the same questions.

The PSOHQ, measuring the satisfaction of the employee with occupational health service and the OP in general, was completed by 89 employees in the intervention group and 63 employees of the control group. Table 2 shows the mean scores of the intervention and control group on the different scales of the PSOHQ. The control group was significantly more satisfied than the intervention group on the following scales: being taken seriously as a patient, expectations and attitude of the employee towards the OHS.

**Table 2:** Results of the Patient Satisfaction with Occupational Health Questionnaire (PSOHQ) (higher scores indicate more satisfaction)

	ECO group (N = 89)	Control group (N = 63)	*P* value
Being taken seriously as a patient	19.7 (4.3)	21.3 (4.4)	.03*
Trust and confidentiality	11.5 (2.2)	12.2 (2.4)	.09
Expectations	9.8 (2.2)	10.7 (2.3)	.02*
Comfort and access	16.5 (3.0)	17.0 (2.4)	.26
Attitude towards the OHS in general	15.6 (4.9)	18.0 (4.8)	<.01*

* Significant at *P* < .05

### Context

In the interviews with the OPs, the OPs reported that they sometimes lost sight of employees. This could be caused by the long time between consultations and the transition of employees to another OP. Due to a reorganization by Arbo Vitale during this study, some of the sick-listed employees were not guided by the same OP all the time, but by different OPs. This was not helpful for the adherence of the OPs to the ECO intervention.

In the interviews with the OPs, they were asked about their opinion of the feasibility of Return@Work in the occupational health setting. According to the OPs, the largest obstacle with regard to the guidance of the OP of Return@Work was the limited time of the OP caused by the low frequency and the short duration of the consultations. An observation of the researchers during the conduct of the study was that it was not possible for employees to contact their OP themselves by telephone outside their regular consultations. This could have caused difficulty when an employee struggled with a module in Return@Work and wanted to ask the OP for advice. During the interviews, one OP suggested using email to support and guide the employee between the regular consultations to increase the contact between the OP and employee.

Finally, this study was conducted in a population in which most of the employees were working in small- to medium-sized companies. Those companies had insurance for the costs of sickness absence and sickness guidance by the OHS that implemented the ECO-intervention, and therefore their motivation for RTW of their employees might have been lower compared to other employers.

## Discussion

The aim of this study was to investigate the feasibility of the blended eHealth intervention, ECO, in the occupational health setting, to report the experiences of the employees and the OPs with the ECO-intervention and to give recommendations for further implementation of the ECO-intervention. The overall results showed that in general the employees and OPs were satisfied with the intervention and the intervention is feasible, however several points could be improved.

In this process evaluation, seven process components were defined. The results of the process components, recruitment and reach, were influenced by the fact that the intervention was implemented during an RCT. The employees could receive Return@Work only if they were willing to participate in a RCT. The requirements of participating in a RCT could have inhibited employees from participating. Furthermore, the training for the OPs in the ECO-intervention was obligatory, which resulted in a reach of 100 %. However, it is possible that the participating OPs were not really motivated to work with the ECO-intervention, and this could be an explanation for the lower OP adherence. It might be interesting for further research to examine the barriers and facilitators for engaging and recruiting OPs for eHealth interventions.

The results of the process components dose delivered and received showed comparable adherence of the employees as in other eHealth interventions [13, 19]. However, the adherence of the employee to the intervention was not optimal. A considerable number of employees did not finish the modules “cognitions with regard to RTW while having symptoms” and “problem-solving skills”. This could have been caused by the fact that these modules were relatively long (respectively four and six sessions) and that the employee could not skip sessions. Another explanation could be that these were the most difficult modules of Return@Work, where the employee might have needed help from the OP, which was provided in only approximately 30 % of cases. The module “relapse prevention” was offered only to the employees who finished the module “problem-solving skills”, and who had (partly) returned to work at that moment. As a consequence, a maximum of four employees may have been offered the module “relapse prevention” and eventually none of them started it. Another explanation for the low number of employees that started this module may be that they did not feel the need for relapse prevention as they were already in the process of returning to work. This is unfortunate because employees with common mental disorders are at increased risk for recurrent sickness absence and relapse prevention could avoid this [20, 21]. Future research may have to pay special attention to the question of how and when relapse prevention should be offered to employees in order to make it attractive and relevant for them to adhere to.

Furthermore, the results on the process component fidelity showed that the support of the OP to the employee in the ECO-intervention was low. It is likely that with more support of the OP, the adherence of the employee to the ECO-intervention could be improved. Thereby maybe also the effectiveness of the ECO-intervention could be improved because eHealth interventions are more effective when they are delivered with human support [22]. A recent study on the role of support in Internet-based problem solving treatment (PST) for symptoms of anxiety and/or depression underscored the importance of structural support in Internet-based interventions [23]. The interviews with the OPs showed that their low involvement in the ECO-intervention was mainly due to the fact that they often were not aware that their employee was participating in the ECO-intervention. This was caused by several reasons. First, as a consequence of the design of the RCT, whereby the recruitment of participants was done by the researchers, the OP was informed about the participation of the employee only when the participant ended the assessment questionnaire in Return@Work. Second, the administrative assistants had to alert the OPs at new email messages in the joint mailbox; this could have caused role-ambiguity by the OPs with the result that they did not feel responsible for being alert at new email-messages. However, halfway through the study the email messages were sent to the OPs directly and still a lot of OPs did not discuss Return@Work with the employees. At last, the unawareness of the OPs could also be a consequence of the fact that the number of employees per OP that participated in the ECO-intervention was low, causing little alertness of the OP to the email messages. It is expected that if in routine practice the recruitment of participants will be done by the professionals that deliver and guide the intervention, the involvement of the professionals will automatically be better.

The results of the process component satisfaction showed that the OPs and employees were satisfied with ECO-intervention, however the employees reported that they had need for more support. The results on the PSOHQ, measuring the satisfaction of the employee with the occupational health service and OP, showed that participants in the control group of the RCT (receiving CAU) were significantly more satisfied than those in the intervention group. The dissatisfaction of the employees in the intervention group with the lack of support of the OP could be an explanation for the lower scores on the PSOHQ. As described above, the support of the OPs was not optimal and could be improved. However, as described by the fidelity and context of the study, the OPs have limited time to support the employees and it is unsure if the regular contacts with the OP are sufficient to support the employees with Return@Work. This could be an important barrier for successful implementation of the ECO-intervention in routine practice. When implementing ECO in the occupational health setting, it is recommended to facilitate the opportunity for the employee to contact the OP, for example by email, outside their regular contacts. Besides, it could be worthwhile to explore the possibilities of studying and implementing the ECO-intervention in other settings, for example primary care.

Finally, in the process component context, it is described that the employers did not have a role in the implementation and intervention in this study. To achieve a successful RTW it is important that all relevant stakeholders facilitate RTW [24]. Perhaps, the implementation and effectiveness of the ECO-intervention could be improved by involving the employers.

### Strengths and Limitations

A strength of this process evaluation is the use of a systematic theoretical framework to report about the several process elements [12]. Another strength is the combination of both qualitative and quantitative data from employees, OPs and the researchers; this gave a detailed view of the feasibility of the ECO-intervention. Finally, the findings from this process evaluation might be useful for several stakeholders when implementing the ECO-intervention.

A limitation of this study is that the interviews with the OPs were conducted halfway through the inclusion period of the RCT; as a consequence the OPs did not have much experience with the ECO-intervention yet. Another limitation is that the opinions of the employees were collected with an open-ended online questionnaire instead of interviews. Interviews had given the opportunity to deepen the information on some themes.

### Implications for Research and Practice

There is a growing emphasis on the importance of including a process evaluation as part of a RCT [15]. We recommend future research on the effectiveness of (eHealth) interventions to perform a process evaluation, because this could narrow the gap between the results of the RCT and implementation in routine practice. When performing a process evaluation it is desirable to use a theoretical framework approach. We used the process components from the framework of Steckler and Linnan [11], however there are a number of domains that can be examined and work is this area is growing [15]. For example, the reviews of Durlak and DuPre and Wierenga et al. both present a summary of different process components and definitions that have been used across process evaluation studies [15, 25].

The results of the RCT of the ECO-intervention showed that ECO led to a faster first RTW and more remission of CMD symptoms 9 months after baseline than CAU [8]. However, no significant effects were found for time to full RTW and remission of symptoms did not persist until 12 months after baseline. This process evaluation showed that the adherence of the employees to the eHealth module, Return@Work, was not optimal and the support of the OP to the employees was lower than anticipated. This indicates that the effectiveness of the ECO-intervention could be further improved. When implementing the ECO-intervention into practice it is recommended to put effort into exploring solutions for improving the adherence of the employees and the support of the OPs.

Finally, literature shows the importance of providing multiple types of supports to stimulate the implementation of innovations [26]. During the implementation of the ECO-intervention the focus was primarily on training and tools (i.e. a manual for the OPs). When implementing ECO it might be helpful to focus on other types of support for example, supervision sessions.

## Conclusion

This process evaluation of the ECO-intervention showed that the intervention seems feasible for further implementation in the occupational health setting, although some barriers need to be addressed. First, the support for the employees by the OPs needs to be facilitated; this could improve the adherence of the employees to and the effectiveness of the ECO-intervention. Second, the involvement of the OPs by the intervention needs to be improved and a solution has to be found for the limited time of the OPs.

A possible solution for both barriers could be extra (telephone) consultations with the OP or the opportunity to contact the OP by email. Because of the feasibility of the ECO-intervention, the satisfaction of the employees and OPs with the ECO-intervention and the positive results of the ECO-intervention on return to work and remission of symptoms [8], the authors recommend to put effort into exploring solutions for the barriers and examining the effectiveness of the ECO-intervention in other settings.

## References

[1] de Graaf R, Tuithof M, van Dorsselaer S, Ten Have M. Sick leave due to psychological and physical illnesses among employees: results of the ‘Netherlands Mental Health Survey and Incidence Study-2’ (NEMESIS-2). [in Dutch: Verzuim door psychische en somatisch aandoeningen bij werkenden: resultaten van de ‘Netherlands Mental Health Survey and Incidence Study-2’ (NEMESIS-2)]. Utrecht: Trimbos-instituut, 2011.

[2] Knudsen AK, Harvey SB, Mykletun A, Overland S (2013). Common mental disorders and long-term sickness absence in a general working population. The Hordaland Health Study. Acta Psychiatr Scand.

[3] Oomens PCJ, Huijs JJHM, Blonk RWB (2010). Effectiveness of the guideline ‘work and psychologial symptoms’ for psychologists (in Dutch: Effectiviteit van de richtlijn ‘Werk en psychische klachten’ voor psychologen).

[4] Anema JR, Van Der Giezen AM, Buijs PC, van Mechelen W (2002). Ineffective disability management by doctors is an obstacle for return-to-work: a cohort study on low back pain patients sicklisted for 3–4 months. Occup Environ Med.

[5] Ejeby K, Savitskij R, Öst L, Ekbom A, Brandt L, Ramnerö J (2014). Symptom reduction due to psychosocial interventions is not accompanied by a reduction in sick leave: results from a randomized controlled trial in primary care. Scand J Prim Health Care.

[6] Nieuwenhuijsen K, Bültmann U, Neumeyer-Gromen A, Verhoeven AC, Verbeek JH, Van der Feltz-Cornelis CM (2008). Interventions to improve occupational health in depressed people. Cochrane Database Syst Rev.

[7] Volker D, Vlasveld MC, Anema JR, Beekman ATF, Hakkaart-van Roijen L, Brouwers EPM (2013). Blended eHealth module on return to work embedded in collaborative occupational health care for common mental disorders: design of a cluster randomized controlled trial. Neuropsychiatr Dis Treat.

[8] Volker D, Zijlstra-Vlasveld MC, Anema JR, Beekman ATF, Brouwers EPM, Emons WHM (2015). Effectiveness of a blended web-based intervention on return to work for sick-listed employees with common mental disorders: results of a cluster randomized controlled trial. J Med Internet Res.

[9] Riper H, Filip S, van der Zanden R, Conijn B, Kramer J, Mutsaers K (2007). E-mental health: high tech, high touch, high trust. Programmeringsstudie E-Mental Health in opdracht van het ministerie van VWS.

[10] Blankers M, Donker T, Riper H (2013). E-mental health in the Netherlands (In Dutch: E-mental health in Nederland). De Psycholoog.

[11] Steckler A, Linnan L, Steckler A, Linnan L (2002). Process evaluation for public health interventions and research. An overview. Process evaluation for public health inteventions and research.

[12] Saunders RP, Evans MH, Joshi P (2005). Developing a process-evaluation plan for assessing health promotion program implementation: a how to guide. Health Promot Pract.

[13] Geraedts AS, Kleiboer AM, Wiezer NM, Cuijpers P, van Mechelen W, Anema JR (2014). Feasibility of a worker-directed web-based intervention for employees with depressive symptoms. Internet Interv.

[14] Andersen LL, Zebis MK (2014). Process evaluation of workplace interventions with physical exercise to reduce musculoskeletel disorders. Int J Rheumatol.

[15] Wierenga D, Engbers LH, van Empelen P, Duijts S, Hildebrandt VH, van Mechelen W (2013). What is actually measured in process evaluations for worksite health promotion programs: a systematic review. BMC Public Health.

[16] Verbeek JH, de Boer AG, van der Weide WE, Piirainen H, Anema JR, Van Amstel RJ (2005). Patient satisfaction with occupational health physicians, development of a questionnaire. Occup Environ Med.

[17] MAXQDA: Qualitative data analysis. Berlin: VERBI software; 2007.

[18] IBM SPSS statistics for windows, version 22.0. Armonk, NY: IBM Corp; 2013.

[19] Bolier L, Haverman M, Kramer J, Westerhof GJ, Riper H, Walburg JA (2013). An internet-based intervention to promote mental fitness for mildly depressed adults: randomized controlled trial. J Med Internet Res.

[20] Koopmans PC, Bültmann U, Roelen CAM, Hoedeman R, van der Klink JJL, Groothoff JW (2011). Recurrence of sickness absence due to common mental disorders. Int Arch Occup Environ Health.

[21] Arends I. Prevention of recurrent sickness absence in workers with common mental disorders (doctoral dissertation). ‘s Hertogenbosch: BOXPress; 2013.

[22] Andersson G, Cuijpers P (2009). Internet-based and other computerized psychological treatments for adult depression: a meta-analysis. Cogn Behav Ther.

[23] Kleiboer A, Donker T, Seekles W, van Straten A, Riper H, Cuijpers P (2015). A randomized controlled trial on the role of support in Internet-based problem solving therapy for depression and anxiety. Behav Res Ther.

[24] van Oostrom SH, Driessen MT, de Vet HCW, Franche RL, Schonstein E, Loisel P (2009). Workplace interventions for preventing work disability (review). Cochrane Database Syst Rev.

[25] Durlak JA, DuPre EP (2008). Implementation matters: a review of research on the influence of implementation on program outcomes and the factors affecting implementation. Am J Community Psychol.

[26] Wandersman A, Chien VH, Katz J (2012). Toward an evidence-based sytstem for innovation support for implementing innovations with quality: tools, training, technical assistance, and quality assurace/quality improvement. Am J Community Psychol.

